# Retrospective Evaluation of the Correlation Between Previous Hospitalizations, the Type of Current Living Space, and Quality of Family Function

**DOI:** 10.3389/fpsyt.2020.00215

**Published:** 2020-03-17

**Authors:** Xiwang Fan, XuDong Zhao, Bingen Zhu, Hongyun Qin

**Affiliations:** Department of Psychiatry, Shanghai Pudong New Area Mental Health Center, Tongji University School of Medicine, Shanghai, China

**Keywords:** previous hospitalizations, current living space, quality of family function, persons living with schizophrenia, community

## Abstract

**Background and Aim:**

There are important public health issues involving rehabilitation of patients living with schizophrenia in the community. The purpose of this study was to analyze the correlations between number of previous hospitalizations, living space and quality of family function in the rehabilitation of patients living with schizophrenia in the community. The study attempts to determine the potential ways to use beneficial factors in recurrent hospitalization for improving treatment and rehabilitation efforts for patients living with long-term chronic schizophrenia.

**Methods:**

The study included 281 rehabilitations of patients living with schizophrenia in the community. A homemade general questionnaire was used to collect information about the number of previous hospitalizations and the living space of the participants. A family assessment device was used to evaluate the quality of family function of the patients.

**Results:**

The number of previous hospitalizations of persons living with schizophrenia in the community was negatively correlated with the quality of family function (*B* = 0.063), and there was no statistical difference in the number of previous hospitalizations and the quality of family function of patients in different living spaces.

**Conclusions:**

The number of previous hospitalizations had a negative impact on family function in the rehabilitation of patients living with schizophrenia in the community. Living space may not have a significant positive effect on family function or the number of previous hospitalizations.

## Introduction

The course of schizophrenia is prolonged, the cure rate is low, the relapse and disability rates are high, and long-term treatment and rehabilitation are needed (1). Ninety percent of the more than 7.8 million persons living with schizophrenia in China live in the community; these people are the main target of community rehabilitation services (2). Persons living with schizophrenia suffer from cognitive impairment and recurrent conditions, so they may be repeatedly hospitalized (3). Some studies have found that an increased number of hospitalizations for persons living with schizophrenia increased the psychological burden on relatives (4). The provision of antipsychotic drugs alone cannot meet the economic, social, and psychological needs of persons living with schizophrenia (5). Clinical researchers believe that the treatment of schizophrenia requires drugs combined with psychological interventions (6). In addition, long-term hospitalization has been shown to greatly impair the social cognitive function of patients (7). Therefore, rehabilitation of persons living with schizophrenia in the community is currently a preferable approach (8).

Studies have shown that people living in densely populated and socially disordered areas are more likely to suffer from mental illness (9). Persons living with schizophrenia who have independent housing have a better quality of life and a better social and emotional network (10). Studies have found that the number of household members and floor space can predict patients' emotional health (11).

The prognosis of persons living with schizophrenia is closely related to their family function (12). Good family function can improve patient compliance and reduce the relapse rate (13). Persons living with schizophrenia have poor family function, insufficient social support, and less communication between family members (14). The family function of persons living with schizophrenia plays an important role in their rehabilitation (15). Good family function can reduce the relapse rate and improve the prognosis of schizophrenia (16). Additionally, family function has a positive regulatory effect on medication compliance in persons living with schizophrenia (17).

Repeated hospitalizations for persons living with schizophrenia will increase the patient's economic and psychological burden and will also increase the cost of government medical insurance (18). The present study analyzed the correlations between the number of hospitalizations of patients rehabilitating in the community, living space, and family function and provided a theoretical basis for reducing the hospitalization rate of persons living with schizophrenia.

## Methods

### Design and Procedures

A cross-sectional study was performed. The study included 281 rehabilitations of patients living with schizophrenia in the community. All participants were from community rehabilitation institutions under the Shanghai Pudong New Area Mental Health Center. The study lasted from May 2018 to August 2018, and sample evaluations were performed by professional clinical researchers.

### Participants

The sample was originally recruited from persons living with schizophrenia in the community rehabilitation facility of the Shanghai Pudong New Area Mental Health Center. The following were the inclusion criteria: (1) met the diagnostic criteria for schizophrenia in the Tenth Edition of the International Statistical Classification of Diseases and Related Health Problems; (2) aged between 18 and 65; (3) were male or female; (4) had no history of head trauma, obvious intellectual disability, or other serious or uncontrolled stable physical illness; (5) had no current use (within the previous 3 months) of alcohol or drugs or a past history of dependence; and (6) had no obvious hallucinations or complaints.

### Measures

A homemade questionnaire collected data on the number of previous hospitalizations and the personal living space of the patients. The number of previous hospitalizations was the number of times a patient was hospitalized in a psychiatric hospital from the first onset of schizophrenia and referred to the patient case for proofreading. According to Chinese traditional culture and customs, people with mental disorders usually live with their families. The personal living space was calculated as the total area of the patient's current house divided by the number of occupants.

### Family Assessment Device

The family assessment device used in this study was a self-report scale that assessed family members' perceptions of family function (19). Previous studies have shown that these family assessment scales have good reliability and validity in the clinic (20). The total score for all items on the scale was the scale score (21). The higher the score was, the better the family structure and function (22). In most clinical studies, this score has a good degree of discrimination (23). Some studies have found that family assessment devices have good adaptability and sensitivity in mainland China (24).

### Statistical Analysis

All results are expressed as the mean ± standard deviation. Statistical analysis was performed using the SPSS 21.0 (SPSS, Chicago, IL, USA) software package. Measurement data that met the normal distribution were compared using independent sample *t*-tests, rank-sum tests were used for data noncompliant with the normal distribution, and one-way ANOVA was used to compare multiple groups. Regression analysis was used to describe the correlations between variables. Statistical correlations between variables in the regression model were determined with *p* < 0.0001.

## Results

**Table 1** is mainly a comparative study of the differences in sex, age, age at onset, and education. The patients of different sexes had significant differences in education (*p* < 0.01). There were no significant differences in the age at onset between males and females.

**Table 1:** General characteristics of the study participants.

Characteristics	All	Male	Female	*p* value
Sex	281	145	136	
Age (years)	44.85 ± 8.92	45.23 ± 9.64	44.45 ± 8.09	0.459
Age at first onset	25.49 ± 8.22	25.29 ± 8.47	25.71 ± 7.99	0.667
Grade				0.008^a^
Elementary school	16	9	7	
Junior high school	117	62	55	
Senior high school	115	58	57	
University education	31	16	15	
Graduate education	2	0	2	

Data are presented as the mean ± SD. ^a^Mann-Whitney U test.

**Table 2** shows that the number of previous hospitalizations, problem solving, communication, affective involvement, and behavior control had significant positive impacts on the general functioning score. However, living space, roles, affective responsiveness, and age at first onset did not affect general functioning.

**Table 2:** General functioning regression analysis results.

	NSC		NC	*t*	*p*	*VIF*	*R* ^2^	Amend *R* ^2^	*F*
	*B*	Standard error	Beta						
Constant	-6.211	1.349	–	-4.604	0.000**	–			
NH	0.063	0.028	0.064	2.225	0.027*	1.011			
LS	-0.284	0.193	-0.044	-1.475	0.141	1.084			
PS	0.825	0.079	0.429	10.378	0.000**	2.063			
CM	0.343	0.063	0.245	5.431	0.000**	2.457			
RO	0.073	0.054	0.056	1.359	0.175	2.068			
AR	0.056	0.081	0.032	0.694	0.488	2.642	0.776	0.769	F(9,271)=104.347,
AI	0.398	0.063	0.234	6.298	0.000**	1.665			P=0.000**
BC	0.24	0.061	0.146	3.933	0.000**	1.677			
AO	0.014	0.016	0.025	0.882	0.379	1.011			
Dependent variable: GF									
D-W value: 2.034									

*Correlation is significant at a confidence level of 0.05; **correlation is significant at a confidence level of 0.0001. The analysis method used was regression analysis.

AI, affective involvement; AO, age at first onset; AR, affective responsiveness; BC, behavior control; CM, communication; GF, general functioning; LS, living space; NC, normalization coefficient; NH, number of previous hospitalizations; NSC, nonstandardized coefficient; PS, problem solving; RO, roles.

According to the analysis results provided in **Table 3**, there were no significant differences in the number of hospitalizations for persons living with schizophrenia in community rehabilitation based on different living spaces.

**Table 3:** Comparative analysis of the number of hospitalizations in different living spaces.

	LS (mean ± SD)				*F*	*p*
	A (n=10)	B (n=91)	C (n=145)	D (n=35)		
NH	1.60 ± 1.65	1.58 ± 1.58	2.61 ± 6.37	1.77 ± 2.51	0.988	0.399

Data are presented as the mean ± SD. The analysis method used was repeated measures ANOVA.

A, less than 10 square meters; B, between 10～20 square meters; C, between 20～40 square meters; D, over 40 square meters; LS, living space; NH, number of previous hospitalizations.

The analysis of variance results in **Table 4** show that there was no statistically significant difference in family function among persons living with schizophrenia based on different living spaces, and there were no significant differences within each factor entry.

**Table 4:** Comparative analysis of family function based on different living spaces.

	LS (mean ± SD)				*F*	*p*
	A (n=10)	B (n=91)	C (n=145)	D (n=35)		
PS	13.30 ± 1.64	12.67 ± 2.23	12.67 ± 2.47	12.31 ± 2.82	0.47	0.703
CM	21.60 ± 2.84	20.10 ± 3.28	19.79 ± 3.32	18.94 ± 3.34	2.015	0.112
RO	27.10 ± 3.84	25.49 ± 3.47	25.17 ± 3.48	25.37 ± 3.97	0.978	0.404
AR	15.70 ± 3.47	13.81 ± 2.45	13.79 ± 2.65	14.20 ± 3.02	1.785	0.15
AI	17.80 ± 2.62	16.57 ± 2.45	16.39 ± 2.77	16.37 ± 3.22	0.888	0.448
BC	20.10 ± 3.38	19.26 ± 2.87	20.00 ± 2.73	20.49 ± 2.86	2.067	0.105
GF	27.40 ± 4.27	24.90 ± 4.64	24.57 ± 4.46	24.09 ± 5.34	1.435	0.233

Data are presented as the mean ± SD. The analysis method used was repeated measures ANOVA.

A, less than 10 square meters; B, between 10～20 square meters; C, between 20～40 square meters; D, over 40 square meters; LS, living space; PS, problem solving; CM, communication; RO, roles; AR, affective responsiveness; AI, affective involvement; BC, behavior control; GF, general functioning.

## Discussion

This study mainly explored the correlations between the number of previous hospitalizations for rehabilitation of patients living with schizophrenia in the community and living space and family function. The study included 281 patients. The difference in educational level between persons living with schizophrenia of different sexes was significant. Sociological surveys have shown that the educational level of women in Shanghai, China, is higher than that of men (25). More than 70% of Shanghai women have a college education or above, and 11.1% of Shanghai women have a graduate education (26). Studies have found that males living with schizophrenia have more cognitive impairments in reasoning and problem solving, social cognition, processing speed, and working memory than females living with schizophrenia (27). The ovarian hormones progesterone and estrogen have good neurotrophic and neuroprotective effects and support reproductive function and cognitive health (28). Estrogen promotes higher cognitive functions by acting in brain regions such as the prefrontal cortex and the hippocampus (29). Therefore, women living with schizophrenia are more likely to continue their education than males with schizophrenia.

The family function behavior control scores were positively related to living space, showing that the larger the living space was, the lower was the behavior control mode. Patients living with schizophrenia in the community who are being rehabilitated have large emotional fluctuations and are prone to have negative emotions (30). Most persons living with schizophrenia cope with problems in a negative and evasive manner (31). Additionally, most persons living with schizophrenia have mild loneliness, and previous research has suggested that solitude makes people feel lonely (32). Therefore, subjects with a large living space may have a single problem-solving mode. The above results indicate that improvements in patient living space alone may not have a significant positive effect on improvements in family function.

There was an inverse correlation between the number of previous hospitalizations for persons living with schizophrenia and family function, such that as the number of previous hospitalizations increased, family function decreased. Previous studies have found that persons living with schizophrenia who were repeatedly hospitalized increased the burden on their families (33). Despite a better public understanding of schizophrenia in recent years, schizophrenia is still widely regarded as shameful for the patients' families and by the Chinese public (34, 35). With the increase in the number of hospitalizations, the stigma around the patients and their families will strengthen (36, 37). The above research results show that the number of previous hospitalizations for rehabilitation of patients living with schizophrenia in the community has a negative effect on family function. This study found that the roles and effective responsiveness scores for the rehabilitation of patients living with schizophrenia in the community were not related to general functioning and may be related to the clinical symptoms of schizophrenia (38, 39).

Shanghai's per capita living space is 36.7 square meters; approximately half of the patients in this study live below the Shanghai average (40). **Table 4** shows that there were no statistically significant differences in the number of previous hospitalizations and family function of the patients based on different living spaces, further indicating that good living spaces had no significant positive effect on the rehabilitation of patients with schizophrenia in the community.

In summary, our study found that the number of previous hospitalizations of persons living with schizophrenia in the community has a negative impact on quality of family function and that there is no statistical difference in the number of previous hospitalizations and quality of family function of patients in different living spaces.

## Data Availability Statement

All datasets generated for this study are included in the article/**Supplementary Material**.

## Ethics Statement

The studies involving human participants were reviewed and approved by the Shanghai Pudong New Area Mental Health Ethics Committee. The patients/participants provided their written informed consent to participate in this study.

## Author Contributions

HQ and XZ contributed to the conception and design of the present study. HQ recruited the patients and conducted the study. XF and BZ undertook the statistical analyses and wrote the first manuscript draft. All authors contributed to the data analysis and drafting and revision of the article, gave final approval of the version to be published, and agreed to be accountable for all aspects of the work.

## Funding

This study was supported by the Shanghai Pudong Municipal Health Commission (PWRd 2017-05 and PWYgy2018-10) and the Shanghai Municipal Health Commission (201840372).

## Conflict of Interest

The authors declare that the research was conducted in the absence of any commercial or financial relationships that could be construed as a potential conflict of interest.
