# Supplementary material for: Retrospective Evaluation of the Correlation Between Previous Hospitalizations, the Type of Current Living Space, and Quality of Family Function
Source: Front Psychiatry. 2020 Mar 17;11:215. doi: 10.3389/fpsyt.2020.00215 (PMC7090217; doi:10.3389/fpsyt.2020.00215)
Supplement: Supplementary file 1 [file DataSheet_1.pdf]

Raw data

| Numbering | Number of previous hospitalizations | Living area | Problem solving | Communication | Roles | Affective responsiveness | Affective involvement | Behavior control | General Functioning | gender | age | Age of first onset |
|---|---|---|---|---|---|---|---|---|---|---|---|---|
| A1 | 25.00 | 3.00 | 12.00 | 24.00 | 25.00 | 17.00 | 17.00 | 18.00 | 26.00 | 1.00 | 55.00 | 25.00 |
| A2 | 1.00 | 3.00 | 11.00 | 18.00 | 26.00 | 12.00 | 16.00 | 20.00 | 24.00 | 2.00 | 46.00 | 29.00 |
| A3 | 3.00 | 4.00 | 16.00 | 23.00 | 32.00 | 17.00 | 19.00 | 23.00 | 29.00 | 1.00 | 50.00 | 26.00 |
| A4 | 2.00 | 2.00 | 14.00 | 20.00 | 31.00 | 17.00 | 20.00 | 22.00 | 26.00 | 1.00 | 47.00 | 30.00 |
| A5 | 3.00 | 3.00 | 13.00 | 18.00 | 23.00 | 12.00 | 15.00 | 20.00 | 25.00 | 1.00 | 39.00 | 24.00 |
| A6 | 6.00 | 2.00 | 13.00 | 17.00 | 19.00 | 13.00 | 13.00 | 17.00 | 18.00 | 1.00 | 46.00 | 30.00 |
| A7 | 3.00 | 2.00 | 16.00 | 23.00 | 31.00 | 17.00 | 18.00 | 24.00 | 31.00 | 2.00 | 46.00 | 33.00 |
| A8 | 5.00 | 3.00 | 11.00 | 21.00 | 25.00 | 12.00 | 15.00 | 20.00 | 25.00 | 2.00 | 53.00 | 53.00 |
| A9 | 0.00 | 3.00 | 11.00 | 17.00 | 16.00 | 13.00 | 16.00 | 16.00 | 21.00 | 1.00 | 30.00 | 13.00 |
| A10 | 3.00 | 2.00 | 10.00 | 19.00 | 23.00 | 12.00 | 15.00 | 17.00 | 23.00 | 1.00 | 46.00 | 16.00 |
| A11 | 3.00 | 3.00 | 15.00 | 20.00 | 29.00 | 17.00 | 15.00 | 20.00 | 23.00 | 1.00 | 42.00 | 18.00 |
| A12 | 0.00 | 3.00 | 14.00 | 22.00 | 29.00 | 13.00 | 16.00 | 19.00 | 27.00 | 2.00 | 40.00 | 16.00 |
| A13 | 1.00 | 2.00 | 15.00 | 29.00 | 26.00 | 20.00 | 18.00 | 23.00 | 34.00 | 2.00 | 34.00 | 19.00 |
| A14 | 0.00 | 2.00 | 12.00 | 18.00 | 21.00 | 12.00 | 18.00 | 19.00 | 24.00 | 2.00 | 33.00 | 18.00 |
| A15 | 2.00 | 2.00 | 16.00 | 25.00 | 29.00 | 16.00 | 20.00 | 18.00 | 29.00 | 2.00 | 56.00 | 28.00 |
| A16 | 0.00 | 2.00 | 12.00 | 17.00 | 26.00 | 14.00 | 18.00 | 21.00 | 25.00 | 2.00 | 57.00 | 28.00 |
| A17 | 5.00 | 2.00 | 11.00 | 17.00 | 30.00 | 12.00 | 20.00 | 19.00 | 21.00 | 2.00 | 51.00 | 20.00 |
| A18 | 1.00 | 2.00 | 14.00 | 20.00 | 24.00 | 13.00 | 15.00 | 16.00 | 27.00 | 1.00 | 39.00 | 16.00 |
| A19 | 1.00 | 2.00 | 13.00 | 19.00 | 26.00 | 12.00 | 18.00 | 19.00 | 26.00 | 2.00 | 37.00 | 22.00 |
| A20 | 1.00 | 2.00 | 11.00 | 19.00 | 25.00 | 14.00 | 16.00 | 18.00 | 24.00 | 2.00 | 44.00 | 21.00 |

| | | | | | | | | | | | | |
|---|---|---|---|---|---|---|---|---|---|---|---|---|
| A21 | 3.00 | 4.00 | 10.00 | 19.00 | 25.00 | 14.00 | 17.00 | 21.00 | 24.00 | 2.00 | 41.00 | 17.00 |
| A22 | 3.00 | 2.00 | 11.00 | 20.00 | 20.00 | 13.00 | 14.00 | 17.00 | 26.00 | 1.00 | 48.00 | 19.00 |
| A23 | 3.00 | 2.00 | 10.00 | 19.00 | 19.00 | 11.00 | 15.00 | 16.00 | 17.00 | 1.00 | 37.00 | 23.00 |
| A24 | 3.00 | 3.00 | 10.00 | 14.00 | 21.00 | 11.00 | 18.00 | 16.00 | 21.00 | 2.00 | 52.00 | 25.00 |
| A25 | 2.00 | 2.00 | 9.00 | 20.00 | 23.00 | 11.00 | 15.00 | 17.00 | 20.00 | 2.00 | 43.00 | 24.00 |
| A26 | 0.00 | 3.00 | 12.00 | 18.00 | 23.00 | 13.00 | 20.00 | 17.00 | 26.00 | 1.00 | 51.00 | 32.00 |
| A27 | 2.00 | 2.00 | 13.00 | 22.00 | 23.00 | 15.00 | 19.00 | 23.00 | 24.00 | 2.00 | 52.00 | 20.00 |
| A28 | 1.00 | 4.00 | 8.00 | 14.00 | 22.00 | 14.00 | 12.00 | 18.00 | 16.00 | 1.00 | 49.00 | 31.00 |
| A29 | 3.00 | 2.00 | 15.00 | 25.00 | 26.00 | 15.00 | 18.00 | 20.00 | 30.00 | 2.00 | 42.00 | 20.00 |
| A30 | 12.00 | 3.00 | 11.00 | 19.00 | 19.00 | 9.00 | 15.00 | 19.00 | 19.00 | 1.00 | 56.00 | 24.00 |
| A31 | 1.00 | 2.00 | 11.00 | 18.00 | 26.00 | 11.00 | 15.00 | 17.00 | 20.00 | 1.00 | 39.00 | 20.00 |
| A32 | 4.00 | 2.00 | 9.00 | 13.00 | 22.00 | 12.00 | 14.00 | 17.00 | 17.00 | 1.00 | 52.00 | 25.00 |
| A33 | 1.00 | 2.00 | 13.00 | 21.00 | 28.00 | 14.00 | 19.00 | 21.00 | 28.00 | 1.00 | 32.00 | 23.00 |
| A34 | 2.00 | 2.00 | 12.00 | 19.00 | 24.00 | 13.00 | 12.00 | 19.00 | 26.00 | 1.00 | 57.00 | 23.00 |
| A35 | 0.00 | 1.00 | 14.00 | 22.00 | 22.00 | 14.00 | 15.00 | 26.00 | 25.00 | 1.00 | 35.00 | 10.00 |
| A36 | 8.00 | 3.00 | 11.00 | 20.00 | 23.00 | 12.00 | 16.00 | 17.00 | 24.00 | 1.00 | 41.00 | 22.00 |
| A37 | 4.00 | 3.00 | 6.00 | 15.00 | 23.00 | 10.00 | 14.00 | 15.00 | 17.00 | 1.00 | 32.00 | 13.00 |
| A38 | 2.00 | 3.00 | 11.00 | 21.00 | 26.00 | 15.00 | 15.00 | 17.00 | 22.00 | 1.00 | 44.00 | 28.00 |
| A39 | 7.00 | 3.00 | 8.00 | 18.00 | 24.00 | 12.00 | 14.00 | 17.00 | 16.00 | 2.00 | 39.00 | 29.00 |
| A40 | 2.00 | 3.00 | 14.00 | 23.00 | 24.00 | 16.00 | 18.00 | 19.00 | 25.00 | 1.00 | 60.00 | 36.00 |
| A41 | 0.00 | 2.00 | 17.00 | 23.00 | 28.00 | 17.00 | 16.00 | 19.00 | 32.00 | 1.00 | 24.00 | 18.00 |
| A42 | 4.00 | 3.00 | 7.00 | 15.00 | 25.00 | 10.00 | 13.00 | 19.00 | 19.00 | 2.00 | 37.00 | 24.00 |
| A43 | 13.00 | 3.00 | 12.00 | 19.00 | 23.00 | 13.00 | 15.00 | 19.00 | 24.00 | 1.00 | 46.00 | 16.00 |
| A44 | 1.00 | 2.00 | 13.00 | 21.00 | 24.00 | 13.00 | 15.00 | 17.00 | 23.00 | 2.00 | 58.00 | 28.00 |

| | | | | | | | | | | | | |
|---|---|---|---|---|---|---|---|---|---|---|---|---|
| A45 | 1.00 | 1.00 | 11.00 | 20.00 | 27.00 | 16.00 | 15.00 | 20.00 | 24.00 | 1.00 | 19.00 | 19.00 |
| A46 | 0.00 | 4.00 | 13.00 | 19.00 | 24.00 | 12.00 | 15.00 | 20.00 | 24.00 | 1.00 | 50.00 | 37.00 |
| A47 | 0.00 | 2.00 | 18.00 | 27.00 | 32.00 | 19.00 | 20.00 | 24.00 | 32.00 | 1.00 | 53.00 | 28.00 |
| A48 | 0.00 | 2.00 | 11.00 | 19.00 | 23.00 | 13.00 | 15.00 | 18.00 | 24.00 | 1.00 | 59.00 | 46.00 |
| A49 | 0.00 | 3.00 | 12.00 | 18.00 | 23.00 | 12.00 | 16.00 | 19.00 | 23.00 | 1.00 | 45.00 | 32.00 |
| A50 | 1.00 | 3.00 | 11.00 | 18.00 | 25.00 | 13.00 | 14.00 | 22.00 | 22.00 | 2.00 | 36.00 | 18.00 |
| A51 | 0.00 | 1.00 | 13.00 | 17.00 | 25.00 | 13.00 | 17.00 | 17.00 | 26.00 | 1.00 | 35.00 | 18.00 |
| A52 | 2.00 | 1.00 | 14.00 | 20.00 | 23.00 | 12.00 | 15.00 | 19.00 | 24.00 | 1.00 | 32.00 | 17.00 |
| A53 | 4.00 | 3.00 | 12.00 | 23.00 | 22.00 | 12.00 | 13.00 | 24.00 | 25.00 | 2.00 | 55.00 | 25.00 |
| A54 | 0.00 | 2.00 | 12.00 | 20.00 | 23.00 | 13.00 | 15.00 | 19.00 | 23.00 | 2.00 | 47.00 | 21.00 |
| A55 | 1.00 | 2.00 | 16.00 | 22.00 | 27.00 | 15.00 | 16.00 | 22.00 | 29.00 | 2.00 | 43.00 | 35.00 |
| A56 | 0.00 | 3.00 | 11.00 | 22.00 | 22.00 | 13.00 | 16.00 | 22.00 | 17.00 | 2.00 | 38.00 | 16.00 |
| A57 | 1.00 | 3.00 | 11.00 | 23.00 | 25.00 | 13.00 | 16.00 | 19.00 | 24.00 | 1.00 | 56.00 | 31.00 |
| A58 | 2.00 | 3.00 | 12.00 | 19.00 | 22.00 | 12.00 | 16.00 | 18.00 | 22.00 | 2.00 | 30.00 | 20.00 |
| A59 | 1.00 | 2.00 | 14.00 | 21.00 | 27.00 | 15.00 | 16.00 | 20.00 | 26.00 | 1.00 | 51.00 | 26.00 |
| A60 | 0.00 | 4.00 | 13.00 | 18.00 | 20.00 | 12.00 | 13.00 | 22.00 | 17.00 | 2.00 | 53.00 | 30.00 |
| A61 | 8.00 | 3.00 | 13.00 | 21.00 | 27.00 | 15.00 | 19.00 | 21.00 | 26.00 | 2.00 | 58.00 | 19.00 |
| A62 | 2.00 | 3.00 | 13.00 | 18.00 | 24.00 | 12.00 | 15.00 | 19.00 | 24.00 | 2.00 | 30.00 | 26.00 |
| A63 | 1.00 | 4.00 | 16.00 | 24.00 | 25.00 | 18.00 | 18.00 | 19.00 | 29.00 | 2.00 | 44.00 | 37.00 |
| A64 | 1.00 | 3.00 | 11.00 | 18.00 | 24.00 | 11.00 | 12.00 | 17.00 | 22.00 | 1.00 | 37.00 | 35.00 |
| A65 | 1.00 | 3.00 | 16.00 | 23.00 | 29.00 | 18.00 | 19.00 | 21.00 | 34.00 | 2.00 | 46.00 | 32.00 |
| A66 | 0.00 | 3.00 | 12.00 | 21.00 | 23.00 | 14.00 | 17.00 | 17.00 | 24.00 | 1.00 | 60.00 | 30.00 |
| A67 | 1.00 | 3.00 | 12.00 | 17.00 | 19.00 | 11.00 | 12.00 | 17.00 | 19.00 | 2.00 | 40.00 | 37.00 |
| A68 | 3.00 | 3.00 | 11.00 | 15.00 | 19.00 | 11.00 | 9.00 | 16.00 | 17.00 | 2.00 | 43.00 | 33.00 |

| | | | | | | | | | | | | |
|---|---|---|---|---|---|---|---|---|---|---|---|---|
| A69 | 1.00 | 3.00 | 10.00 | 16.00 | 21.00 | 12.00 | 12.00 | 17.00 | 20.00 | 2.00 | 40.00 | 21.00 |
| A70 | 0.00 | 3.00 | 11.00 | 18.00 | 28.00 | 12.00 | 17.00 | 21.00 | 23.00 | 2.00 | 51.00 | 40.00 |
| A71 | 2.00 | 2.00 | 9.00 | 13.00 | 16.00 | 9.00 | 10.00 | 13.00 | 12.00 | 2.00 | 47.00 | 16.00 |
| A72 | 3.00 | 3.00 | 11.00 | 13.00 | 21.00 | 12.00 | 14.00 | 20.00 | 17.00 | 2.00 | 45.00 | 34.00 |
| A73 | 10.00 | 3.00 | 10.00 | 17.00 | 20.00 | 10.00 | 13.00 | 17.00 | 19.00 | 1.00 | 60.00 | 22.00 |
| A74 | 0.00 | 3.00 | 13.00 | 19.00 | 21.00 | 15.00 | 14.00 | 14.00 | 17.00 | 1.00 | 36.00 | 21.00 |
| A75 | 3.00 | 3.00 | 13.00 | 22.00 | 25.00 | 14.00 | 15.00 | 20.00 | 26.00 | 2.00 | 49.00 | 18.00 |
| A76 | 1.00 | 3.00 | 13.00 | 21.00 | 27.00 | 15.00 | 18.00 | 24.00 | 25.00 | 1.00 | 48.00 | 28.00 |
| A77 | 4.00 | 3.00 | 9.00 | 15.00 | 17.00 | 10.00 | 17.00 | 24.00 | 20.00 | 1.00 | 50.00 | 34.00 |
| A78 | 2.00 | 2.00 | 12.00 | 20.00 | 25.00 | 13.00 | 15.00 | 18.00 | 23.00 | 2.00 | 24.00 | 13.00 |
| A79 | 3.00 | 3.00 | 16.00 | 24.00 | 22.00 | 13.00 | 18.00 | 21.00 | 29.00 | 1.00 | 48.00 | 18.00 |
| A80 | 5.00 | 3.00 | 9.00 | 15.00 | 24.00 | 9.00 | 14.00 | 18.00 | 16.00 | 1.00 | 45.00 | 18.00 |
| A81 | 0.00 | 3.00 | 14.00 | 17.00 | 24.00 | 12.00 | 14.00 | 20.00 | 25.00 | 2.00 | 46.00 | 19.00 |
| A82 | 1.00 | 2.00 | 11.00 | 20.00 | 22.00 | 14.00 | 12.00 | 16.00 | 22.00 | 1.00 | 58.00 | 42.00 |
| A83 | 4.00 | 2.00 | 10.00 | 21.00 | 30.00 | 16.00 | 25.00 | 25.00 | 28.00 | 2.00 | 37.00 | 17.00 |
| A84 | 0.00 | 2.00 | 12.00 | 18.00 | 20.00 | 11.00 | 15.00 | 20.00 | 21.00 | 2.00 | 33.00 | 16.00 |
| A85 | 0.00 | 4.00 | 14.00 | 18.00 | 23.00 | 13.00 | 15.00 | 24.00 | 26.00 | 2.00 | 49.00 | 18.00 |
| A86 | 3.00 | 3.00 | 13.00 | 17.00 | 18.00 | 11.00 | 15.00 | 20.00 | 23.00 | 2.00 | 49.00 | 37.00 |
| A87 | 1.00 | 3.00 | 12.00 | 20.00 | 30.00 | 16.00 | 18.00 | 21.00 | 26.00 | 1.00 | 59.00 | 47.00 |
| A88 | 0.00 | 3.00 | 8.00 | 12.00 | 18.00 | 10.00 | 14.00 | 18.00 | 16.00 | 2.00 | 37.00 | 31.00 |
| A89 | 2.00 | 3.00 | 13.00 | 24.00 | 28.00 | 14.00 | 22.00 | 21.00 | 26.00 | 1.00 | 31.00 | 18.00 |
| A90 | 0.00 | 3.00 | 12.00 | 18.00 | 25.00 | 12.00 | 16.00 | 19.00 | 24.00 | 1.00 | 46.00 | 20.00 |
| A91 | 1.00 | 4.00 | 12.00 | 20.00 | 23.00 | 13.00 | 15.00 | 19.00 | 26.00 | 1.00 | 33.00 | 26.00 |
| A92 | 0.00 | 3.00 | 13.00 | 20.00 | 25.00 | 16.00 | 19.00 | 22.00 | 26.00 | 1.00 | 31.00 | 20.00 |

| | | | | | | | | | | | | |
|---|---|---|---|---|---|---|---|---|---|---|---|---|
| A93 | 3.00 | 2.00 | 14.00 | 16.00 | 26.00 | 9.00 | 16.00 | 19.00 | 23.00 | 1.00 | 55.00 | 33.00 |
| A94 | 10.00 | 4.00 | 12.00 | 22.00 | 36.00 | 21.00 | 25.00 | 22.00 | 27.00 | 1.00 | 55.00 | 17.00 |
| A95 | 5.00 | 4.00 | 15.00 | 23.00 | 24.00 | 14.00 | 13.00 | 21.00 | 24.00 | 2.00 | 52.00 | 22.00 |
| A96 | 4.00 | 3.00 | 12.00 | 19.00 | 25.00 | 12.00 | 17.00 | 18.00 | 24.00 | 1.00 | 36.00 | 19.00 |
| A97 | 2.00 | 1.00 | 11.00 | 24.00 | 34.00 | 14.00 | 20.00 | 20.00 | 29.00 | 1.00 | 30.00 | 20.00 |
| A98 | 0.00 | 3.00 | 12.00 | 14.00 | 30.00 | 11.00 | 18.00 | 17.00 | 18.00 | 1.00 | 60.00 | 36.00 |
| A99 | 1.00 | 3.00 | 10.00 | 19.00 | 23.00 | 14.00 | 16.00 | 19.00 | 23.00 | 2.00 | 34.00 | 17.00 |
| A100 | 2.00 | 2.00 | 12.00 | 18.00 | 26.00 | 12.00 | 17.00 | 18.00 | 24.00 | 1.00 | 45.00 | 23.00 |
| A101 | 0.00 | 3.00 | 12.00 | 21.00 | 23.00 | 12.00 | 16.00 | 18.00 | 25.00 | 2.00 | 43.00 | 16.00 |
| A102 | 4.00 | 2.00 | 12.00 | 23.00 | 29.00 | 15.00 | 18.00 | 20.00 | 24.00 | 2.00 | 46.00 | 30.00 |
| A103 | 1.00 | 3.00 | 19.00 | 18.00 | 32.00 | 16.00 | 13.00 | 24.00 | 27.00 | 2.00 | 45.00 | 18.00 |
| A104 | 2.00 | 3.00 | 18.00 | 21.00 | 25.00 | 16.00 | 18.00 | 19.00 | 31.00 | 1.00 | 51.00 | 15.00 |
| A105 | 0.00 | 3.00 | 17.00 | 24.00 | 28.00 | 15.00 | 18.00 | 21.00 | 26.00 | 1.00 | 60.00 | 24.00 |
| A106 | 2.00 | 4.00 | 12.00 | 17.00 | 27.00 | 14.00 | 20.00 | 18.00 | 24.00 | 2.00 | 48.00 | 35.00 |
| A107 | 0.00 | 4.00 | 15.00 | 19.00 | 27.00 | 15.00 | 16.00 | 23.00 | 28.00 | 1.00 | 58.00 | 21.00 |
| A108 | 1.00 | 4.00 | 15.00 | 21.00 | 28.00 | 12.00 | 15.00 | 21.00 | 27.00 | 2.00 | 51.00 | 21.00 |
| A109 | 1.00 | 2.00 | 11.00 | 20.00 | 23.00 | 11.00 | 16.00 | 18.00 | 24.00 | 2.00 | 55.00 | 33.00 |
| A110 | 1.00 | 2.00 | 12.00 | 18.00 | 23.00 | 12.00 | 17.00 | 19.00 | 25.00 | 1.00 | 38.00 | 16.00 |
| A111 | 2.00 | 2.00 | 12.00 | 18.00 | 25.00 | 12.00 | 16.00 | 18.00 | 24.00 | 2.00 | 54.00 | 41.00 |
| A112 | 1.00 | 4.00 | 12.00 | 19.00 | 25.00 | 12.00 | 17.00 | 20.00 | 24.00 | 1.00 | 34.00 | 16.00 |
| A113 | 1.00 | 3.00 | 11.00 | 19.00 | 24.00 | 13.00 | 14.00 | 20.00 | 23.00 | 2.00 | 53.00 | 26.00 |
| A114 | 2.00 | 4.00 | 11.00 | 18.00 | 30.00 | 14.00 | 17.00 | 24.00 | 26.00 | 2.00 | 45.00 | 21.00 |
| A115 | 0.00 | 2.00 | 17.00 | 24.00 | 28.00 | 18.00 | 18.00 | 22.00 | 34.00 | 1.00 | 37.00 | 17.00 |
| A116 | 1.00 | 3.00 | 14.00 | 22.00 | 27.00 | 16.00 | 21.00 | 22.00 | 29.00 | 2.00 | 41.00 | 25.00 |

| | | | | | | | | | | | | |
|---|---|---|---|---|---|---|---|---|---|---|---|---|
| A117 | 0.00 | 4.00 | 13.00 | 18.00 | 23.00 | 13.00 | 15.00 | 20.00 | 25.00 | 1.00 | 45.00 | 25.00 |
| A118 | 2.00 | 3.00 | 15.00 | 24.00 | 31.00 | 19.00 | 19.00 | 20.00 | 30.00 | 1.00 | 50.00 | 26.00 |
| A119 | 2.00 | 4.00 | 12.00 | 17.00 | 25.00 | 15.00 | 15.00 | 24.00 | 23.00 | 2.00 | 56.00 | 25.00 |
| A120 | 1.00 | 2.00 | 13.00 | 18.00 | 27.00 | 13.00 | 17.00 | 19.00 | 24.00 | 1.00 | 59.00 | 18.00 |
| A121 | 2.00 | 2.00 | 13.00 | 20.00 | 29.00 | 12.00 | 18.00 | 17.00 | 25.00 | 1.00 | 55.00 | 43.00 |
| A122 | 1.00 | 2.00 | 13.00 | 22.00 | 32.00 | 16.00 | 16.00 | 20.00 | 28.00 | 2.00 | 37.00 | 21.00 |
| A123 | 3.00 | 2.00 | 15.00 | 23.00 | 29.00 | 17.00 | 20.00 | 22.00 | 33.00 | 1.00 | 49.00 | 27.00 |
| A124 | 0.00 | 2.00 | 11.00 | 19.00 | 22.00 | 12.00 | 14.00 | 13.00 | 17.00 | 2.00 | 58.00 | 30.00 |
| A125 | 1.00 | 2.00 | 12.00 | 19.00 | 27.00 | 12.00 | 17.00 | 19.00 | 22.00 | 2.00 | 39.00 | 22.00 |
| A126 | 3.00 | 2.00 | 8.00 | 13.00 | 17.00 | 9.00 | 12.00 | 13.00 | 17.00 | 1.00 | 39.00 | 15.00 |
| A127 | 3.00 | 2.00 | 9.00 | 17.00 | 24.00 | 10.00 | 16.00 | 15.00 | 20.00 | 1.00 | 42.00 | 20.00 |
| A128 | 2.00 | 3.00 | 12.00 | 18.00 | 24.00 | 12.00 | 17.00 | 18.00 | 21.00 | 1.00 | 45.00 | 33.00 |
| A129 | 3.00 | 2.00 | 13.00 | 21.00 | 23.00 | 13.00 | 14.00 | 19.00 | 24.00 | 1.00 | 46.00 | 26.00 |
| A130 | 1.00 | 4.00 | 12.00 | 21.00 | 26.00 | 18.00 | 16.00 | 24.00 | 26.00 | 2.00 | 49.00 | 33.00 |
| A131 | 4.00 | 4.00 | 6.00 | 12.00 | 22.00 | 6.00 | 15.00 | 13.00 | 12.00 | 1.00 | 37.00 | 22.00 |
| A132 | 2.00 | 2.00 | 13.00 | 18.00 | 29.00 | 12.00 | 17.00 | 19.00 | 23.00 | 2.00 | 42.00 | 23.00 |
| A133 | 0.00 | 3.00 | 13.00 | 19.00 | 23.00 | 12.00 | 16.00 | 18.00 | 21.00 | 2.00 | 33.00 | 18.00 |
| A134 | 2.00 | 2.00 | 11.00 | 18.00 | 23.00 | 12.00 | 13.00 | 17.00 | 17.00 | 2.00 | 44.00 | 24.00 |
| A135 | 0.00 | 2.00 | 13.00 | 25.00 | 26.00 | 15.00 | 17.00 | 22.00 | 23.00 | 1.00 | 60.00 | 21.00 |
| A136 | 2.00 | 3.00 | 17.00 | 26.00 | 28.00 | 18.00 | 19.00 | 22.00 | 34.00 | 2.00 | 50.00 | 19.00 |
| A137 | 1.00 | 3.00 | 12.00 | 17.00 | 23.00 | 11.00 | 18.00 | 20.00 | 21.00 | 2.00 | 50.00 | 20.00 |
| A138 | 0.00 | 2.00 | 13.00 | 20.00 | 28.00 | 13.00 | 17.00 | 21.00 | 22.00 | 2.00 | 48.00 | 29.00 |
| A139 | 5.00 | 2.00 | 12.00 | 21.00 | 23.00 | 16.00 | 15.00 | 18.00 | 26.00 | 1.00 | 42.00 | 30.00 |
| A140 | 3.00 | 1.00 | 15.00 | 23.00 | 32.00 | 17.00 | 18.00 | 19.00 | 34.00 | 2.00 | 41.00 | 21.00 |

| | | | | | | | | | | | | |
|---|---|---|---|---|---|---|---|---|---|---|---|---|
| A141 | 0.00 | 2.00 | 12.00 | 20.00 | 25.00 | 12.00 | 17.00 | 16.00 | 24.00 | 1.00 | 59.00 | 24.00 |
| A142 | 3.00 | 3.00 | 14.00 | 24.00 | 26.00 | 15.00 | 15.00 | 21.00 | 25.00 | 1.00 | 48.00 | 16.00 |
| A143 | 0.00 | 3.00 | 12.00 | 21.00 | 34.00 | 18.00 | 24.00 | 25.00 | 29.00 | 1.00 | 49.00 | 17.00 |
| A144 | 1.00 | 2.00 | 12.00 | 17.00 | 23.00 | 10.00 | 14.00 | 17.00 | 22.00 | 2.00 | 55.00 | 37.00 |
| A145 | 0.00 | 2.00 | 13.00 | 18.00 | 30.00 | 12.00 | 16.00 | 19.00 | 24.00 | 2.00 | 48.00 | 25.00 |
| A146 | 0.00 | 3.00 | 12.00 | 22.00 | 25.00 | 12.00 | 15.00 | 18.00 | 24.00 | 2.00 | 43.00 | 20.00 |
| A147 | 0.00 | 4.00 | 16.00 | 24.00 | 31.00 | 18.00 | 18.00 | 23.00 | 34.00 | 1.00 | 59.00 | 41.00 |
| A148 | 0.00 | 3.00 | 12.00 | 20.00 | 25.00 | 12.00 | 14.00 | 18.00 | 24.00 | 2.00 | 50.00 | 32.00 |
| A149 | 0.00 | 2.00 | 14.00 | 23.00 | 26.00 | 16.00 | 15.00 | 20.00 | 27.00 | 1.00 | 58.00 | 20.00 |
| A150 | 1.00 | 2.00 | 11.00 | 18.00 | 22.00 | 12.00 | 14.00 | 13.00 | 19.00 | 2.00 | 48.00 | 32.00 |
| A151 | 0.00 | 2.00 | 14.00 | 24.00 | 28.00 | 16.00 | 19.00 | 16.00 | 32.00 | 2.00 | 31.00 | 25.00 |
| A152 | 0.00 | 3.00 | 12.00 | 19.00 | 27.00 | 12.00 | 16.00 | 20.00 | 26.00 | 2.00 | 48.00 | 38.00 |
| A153 | 1.00 | 3.00 | 13.00 | 22.00 | 29.00 | 15.00 | 18.00 | 22.00 | 29.00 | 1.00 | 34.00 | 17.00 |
| A154 | 0.00 | 4.00 | 9.00 | 18.00 | 29.00 | 12.00 | 24.00 | 26.00 | 29.00 | 2.00 | 43.00 | 29.00 |
| A155 | 0.00 | 2.00 | 12.00 | 19.00 | 25.00 | 14.00 | 17.00 | 20.00 | 24.00 | 2.00 | 60.00 | 48.00 |
| A156 | 0.00 | 3.00 | 14.00 | 20.00 | 28.00 | 14.00 | 18.00 | 21.00 | 27.00 | 2.00 | 37.00 | 26.00 |
| A157 | 0.00 | 3.00 | 12.00 | 18.00 | 29.00 | 12.00 | 17.00 | 20.00 | 24.00 | 1.00 | 47.00 | 26.00 |
| A158 | 8.00 | 3.00 | 14.00 | 25.00 | 25.00 | 17.00 | 15.00 | 19.00 | 26.00 | 1.00 | 52.00 | 25.00 |
| A159 | 0.00 | 3.00 | 6.00 | 12.00 | 20.00 | 6.00 | 13.00 | 13.00 | 14.00 | 2.00 | 48.00 | 29.00 |
| A160 | 1.00 | 3.00 | 14.00 | 24.00 | 24.00 | 16.00 | 19.00 | 21.00 | 30.00 | 2.00 | 30.00 | 28.00 |
| A161 | 1.00 | 3.00 | 16.00 | 27.00 | 31.00 | 17.00 | 19.00 | 24.00 | 33.00 | 1.00 | 57.00 | 43.00 |
| A162 | 0.00 | 3.00 | 14.00 | 24.00 | 25.00 | 20.00 | 17.00 | 25.00 | 29.00 | 1.00 | 40.00 | 22.00 |
| A163 | 0.00 | 2.00 | 13.00 | 23.00 | 25.00 | 13.00 | 20.00 | 22.00 | 27.00 | 1.00 | 27.00 | 25.00 |
| A164 | 0.00 | 4.00 | 12.00 | 19.00 | 24.00 | 13.00 | 14.00 | 19.00 | 24.00 | 1.00 | 58.00 | 45.00 |

| | | | | | | | | | | | | |
|---|---|---|---|---|---|---|---|---|---|---|---|---|
| A165 | 8.00 | 2.00 | 11.00 | 21.00 | 25.00 | 16.00 | 17.00 | 23.00 | 23.00 | 1.00 | 36.00 | 22.00 |
| A166 | 4.00 | 3.00 | 14.00 | 24.00 | 27.00 | 15.00 | 15.00 | 20.00 | 25.00 | 2.00 | 58.00 | 25.00 |
| A167 | 0.00 | 3.00 | 13.00 | 23.00 | 27.00 | 16.00 | 20.00 | 23.00 | 28.00 | 2.00 | 53.00 | 26.00 |
| A168 | 2.00 | 2.00 | 12.00 | 16.00 | 22.00 | 13.00 | 20.00 | 19.00 | 22.00 | 2.00 | 40.00 | 31.00 |
| A169 | 1.00 | 2.00 | 12.00 | 21.00 | 25.00 | 13.00 | 15.00 | 16.00 | 25.00 | 1.00 | 23.00 | 17.00 |
| A170 | 0.00 | 3.00 | 13.00 | 23.00 | 30.00 | 16.00 | 21.00 | 24.00 | 28.00 | 2.00 | 40.00 | 22.00 |
| A171 | 0.00 | 3.00 | 14.00 | 20.00 | 28.00 | 14.00 | 19.00 | 26.00 | 28.00 | 2.00 | 58.00 | 41.00 |
| A172 | 1.00 | 3.00 | 13.00 | 19.00 | 23.00 | 13.00 | 15.00 | 19.00 | 24.00 | 2.00 | 47.00 | 20.00 |
| A173 | 0.00 | 2.00 | 15.00 | 20.00 | 30.00 | 18.00 | 17.00 | 24.00 | 29.00 | 2.00 | 42.00 | 34.00 |
| A174 | 0.00 | 3.00 | 14.00 | 18.00 | 28.00 | 15.00 | 19.00 | 22.00 | 26.00 | 1.00 | 46.00 | 23.00 |
| A175 | 0.00 | 1.00 | 14.00 | 22.00 | 27.00 | 17.00 | 17.00 | 26.00 | 27.00 | 1.00 | 26.00 | 24.00 |
| A176 | 1.00 | 2.00 | 15.00 | 25.00 | 31.00 | 16.00 | 16.00 | 24.00 | 30.00 | 1.00 | 58.00 | 55.00 |
| A177 | 0.00 | 3.00 | 8.00 | 15.00 | 21.00 | 13.00 | 17.00 | 21.00 | 20.00 | 2.00 | 35.00 | 15.00 |
| A178 | 2.00 | 3.00 | 21.00 | 24.00 | 21.00 | 12.00 | 7.00 | 18.00 | 30.00 | 1.00 | 57.00 | 39.00 |
| A179 | 0.00 | 2.00 | 14.00 | 20.00 | 27.00 | 14.00 | 16.00 | 23.00 | 27.00 | 1.00 | 45.00 | 35.00 |
| A180 | 1.00 | 2.00 | 12.00 | 19.00 | 24.00 | 13.00 | 14.00 | 20.00 | 24.00 | 1.00 | 57.00 | 25.00 |
| A181 | 1.00 | 2.00 | 13.00 | 21.00 | 26.00 | 15.00 | 16.00 | 22.00 | 25.00 | 1.00 | 32.00 | 25.00 |
| A182 | 0.00 | 3.00 | 14.00 | 18.00 | 28.00 | 14.00 | 18.00 | 24.00 | 25.00 | 2.00 | 47.00 | 35.00 |
| A183 | 4.00 | 3.00 | 12.00 | 19.00 | 23.00 | 14.00 | 14.00 | 20.00 | 24.00 | 2.00 | 35.00 | 27.00 |
| A184 | 0.00 | 2.00 | 13.00 | 22.00 | 27.00 | 14.00 | 17.00 | 22.00 | 26.00 | 1.00 | 39.00 | 17.00 |
| A185 | 1.00 | 3.00 | 17.00 | 26.00 | 27.00 | 18.00 | 16.00 | 24.00 | 34.00 | 1.00 | 38.00 | 22.00 |
| A186 | 1.00 | 3.00 | 15.00 | 22.00 | 29.00 | 14.00 | 17.00 | 24.00 | 26.00 | 1.00 | 50.00 | 37.00 |
| A187 | 1.00 | 4.00 | 15.00 | 25.00 | 24.00 | 17.00 | 18.00 | 18.00 | 32.00 | 1.00 | 57.00 | 46.00 |
| A188 | 2.00 | 3.00 | 10.00 | 21.00 | 26.00 | 14.00 | 17.00 | 22.00 | 23.00 | 2.00 | 46.00 | 16.00 |

| | | | | | | | | | | | | |
|---|---|---|---|---|---|---|---|---|---|---|---|---|
| A189 | 4.00 | 3.00 | 14.00 | 20.00 | 25.00 | 15.00 | 17.00 | 20.00 | 26.00 | 2.00 | 42.00 | 21.00 |
| A190 | 3.00 | 3.00 | 18.00 | 24.00 | 28.00 | 12.00 | 8.00 | 28.00 | 30.00 | 2.00 | 35.00 | 21.00 |
| A191 | 2.00 | 2.00 | 12.00 | 20.00 | 25.00 | 13.00 | 14.00 | 19.00 | 25.00 | 2.00 | 50.00 | 24.00 |
| A192 | 1.00 | 3.00 | 12.00 | 25.00 | 30.00 | 16.00 | 21.00 | 21.00 | 26.00 | 1.00 | 43.00 | 37.00 |
| A193 | 7.00 | 4.00 | 16.00 | 19.00 | 32.00 | 16.00 | 21.00 | 24.00 | 33.00 | 2.00 | 60.00 | 27.00 |
| A194 | 2.00 | 3.00 | 10.00 | 19.00 | 20.00 | 12.00 | 15.00 | 21.00 | 20.00 | 1.00 | 33.00 | 25.00 |
| A195 | 0.00 | 3.00 | 14.00 | 19.00 | 27.00 | 14.00 | 15.00 | 19.00 | 22.00 | 1.00 | 51.00 | 28.00 |
| A196 | 1.00 | 3.00 | 12.00 | 18.00 | 24.00 | 13.00 | 17.00 | 19.00 | 27.00 | 2.00 | 38.00 | 22.00 |
| A197 | 4.00 | 3.00 | 12.00 | 20.00 | 23.00 | 12.00 | 14.00 | 20.00 | 24.00 | 2.00 | 38.00 | 23.00 |
| A198 | 0.00 | 2.00 | 15.00 | 20.00 | 29.00 | 18.00 | 14.00 | 21.00 | 28.00 | 1.00 | 39.00 | 30.00 |
| A199 | 0.00 | 2.00 | 11.00 | 17.00 | 25.00 | 12.00 | 13.00 | 17.00 | 19.00 | 1.00 | 52.00 | 30.00 |
| A200 | 1.00 | 2.00 | 7.00 | 14.00 | 26.00 | 11.00 | 19.00 | 17.00 | 17.00 | 2.00 | 45.00 | 39.00 |
| A201 | 0.00 | 3.00 | 9.00 | 26.00 | 28.00 | 19.00 | 21.00 | 24.00 | 19.00 | 1.00 | 56.00 | 28.00 |
| A202 | 2.00 | 3.00 | 13.00 | 19.00 | 26.00 | 12.00 | 14.00 | 18.00 | 25.00 | 1.00 | 44.00 | 22.00 |
| A203 | 2.00 | 3.00 | 12.00 | 17.00 | 28.00 | 16.00 | 17.00 | 21.00 | 26.00 | 1.00 | 50.00 | 26.00 |
| A204 | 4.00 | 3.00 | 15.00 | 14.00 | 28.00 | 12.00 | 18.00 | 18.00 | 18.00 | 1.00 | 42.00 | 24.00 |
| A205 | 0.00 | 3.00 | 14.00 | 18.00 | 25.00 | 16.00 | 19.00 | 23.00 | 27.00 | 1.00 | 29.00 | 20.00 |
| A206 | 1.00 | 4.00 | 11.00 | 18.00 | 23.00 | 13.00 | 14.00 | 20.00 | 23.00 | 1.00 | 53.00 | 32.00 |
| A207 | 3.00 | 3.00 | 12.00 | 26.00 | 25.00 | 16.00 | 19.00 | 26.00 | 31.00 | 1.00 | 55.00 | 35.00 |
| A208 | 1.00 | 2.00 | 9.00 | 12.00 | 21.00 | 15.00 | 17.00 | 19.00 | 23.00 | 1.00 | 38.00 | 21.00 |
| A209 | 2.00 | 3.00 | 8.00 | 20.00 | 24.00 | 11.00 | 12.00 | 16.00 | 15.00 | 1.00 | 55.00 | 43.00 |
| A210 | 0.00 | 3.00 | 16.00 | 20.00 | 29.00 | 17.00 | 17.00 | 20.00 | 30.00 | 1.00 | 46.00 | 23.00 |
| A211 | 1.00 | 3.00 | 13.00 | 20.00 | 24.00 | 11.00 | 15.00 | 16.00 | 22.00 | 2.00 | 33.00 | 20.00 |
| A212 | 2.00 | 3.00 | 11.00 | 19.00 | 29.00 | 18.00 | 22.00 | 18.00 | 25.00 | 1.00 | 32.00 | 17.00 |

| | | | | | | | | | | | | |
|---|---|---|---|---|---|---|---|---|---|---|---|---|
| A213 | 2.00 | 3.00 | 16.00 | 21.00 | 24.00 | 19.00 | 19.00 | 25.00 | 36.00 | 1.00 | 35.00 | 20.00 |
| A214 | 0.00 | 3.00 | 8.00 | 14.00 | 18.00 | 11.00 | 14.00 | 15.00 | 15.00 | 2.00 | 35.00 | 22.00 |
| A215 | 1.00 | 4.00 | 12.00 | 12.00 | 19.00 | 10.00 | 15.00 | 14.00 | 17.00 | 1.00 | 35.00 | 18.00 |
| A216 | 0.00 | 3.00 | 15.00 | 21.00 | 27.00 | 14.00 | 21.00 | 21.00 | 28.00 | 2.00 | 46.00 | 26.00 |
| A217 | 0.00 | 3.00 | 12.00 | 20.00 | 23.00 | 12.00 | 16.00 | 20.00 | 24.00 | 2.00 | 37.00 | 19.00 |
| A218 | 1.00 | 2.00 | 13.00 | 20.00 | 25.00 | 13.00 | 17.00 | 19.00 | 26.00 | 2.00 | 52.00 | 24.00 |
| A219 | 2.00 | 3.00 | 13.00 | 19.00 | 27.00 | 12.00 | 18.00 | 18.00 | 25.00 | 2.00 | 43.00 | 16.00 |
| A220 | 0.00 | 4.00 | 13.00 | 19.00 | 27.00 | 12.00 | 16.00 | 20.00 | 24.00 | 2.00 | 46.00 | 21.00 |
| A221 | 1.00 | 2.00 | 13.00 | 21.00 | 26.00 | 15.00 | 18.00 | 19.00 | 28.00 | 2.00 | 52.00 | 50.00 |
| A222 | 0.00 | 4.00 | 13.00 | 17.00 | 26.00 | 15.00 | 16.00 | 18.00 | 23.00 | 1.00 | 53.00 | 34.00 |
| A223 | 4.00 | 3.00 | 13.00 | 22.00 | 25.00 | 15.00 | 17.00 | 18.00 | 26.00 | 2.00 | 33.00 | 25.00 |
| A224 | 3.00 | 3.00 | 13.00 | 20.00 | 28.00 | 18.00 | 16.00 | 21.00 | 24.00 | 1.00 | 46.00 | 21.00 |
| A225 | 2.00 | 3.00 | 13.00 | 17.00 | 24.00 | 17.00 | 15.00 | 20.00 | 19.00 | 2.00 | 60.00 | 34.00 |
| A226 | 0.00 | 4.00 | 9.00 | 14.00 | 24.00 | 18.00 | 14.00 | 22.00 | 16.00 | 1.00 | 39.00 | 17.00 |
| A227 | 2.00 | 3.00 | 15.00 | 24.00 | 36.00 | 15.00 | 15.00 | 22.00 | 32.00 | 2.00 | 45.00 | 30.00 |
| A228 | 1.00 | 3.00 | 12.00 | 18.00 | 24.00 | 13.00 | 18.00 | 21.00 | 25.00 | 2.00 | 40.00 | 25.00 |
| A229 | 70.00 | 3.00 | 14.00 | 19.00 | 25.00 | 13.00 | 14.00 | 22.00 | 31.00 | 2.00 | 54.00 | 36.00 |
| A230 | 0.00 | 3.00 | 11.00 | 16.00 | 21.00 | 12.00 | 15.00 | 18.00 | 16.00 | 2.00 | 29.00 | 16.00 |
| A231 | 4.00 | 3.00 | 15.00 | 26.00 | 28.00 | 17.00 | 17.00 | 22.00 | 32.00 | 1.00 | 36.00 | 20.00 |
| A232 | 4.00 | 2.00 | 15.00 | 27.00 | 27.00 | 18.00 | 18.00 | 25.00 | 31.00 | 1.00 | 47.00 | 23.00 |
| A233 | 0.00 | 3.00 | 14.00 | 20.00 | 25.00 | 14.00 | 19.00 | 21.00 | 26.00 | 2.00 | 44.00 | 18.00 |
| A234 | 1.00 | 2.00 | 14.00 | 21.00 | 28.00 | 16.00 | 20.00 | 18.00 | 25.00 | 2.00 | 49.00 | 30.00 |
| A235 | 2.00 | 3.00 | 12.00 | 19.00 | 26.00 | 13.00 | 21.00 | 20.00 | 26.00 | 1.00 | 36.00 | 21.00 |
| A236 | 3.00 | 3.00 | 14.00 | 21.00 | 28.00 | 16.00 | 19.00 | 21.00 | 25.00 | 2.00 | 50.00 | 25.00 |

| | | | | | | | | | | | | |
|---|---|---|---|---|---|---|---|---|---|---|---|---|
| A237 | 0.00 | 3.00 | 14.00 | 23.00 | 30.00 | 18.00 | 19.00 | 19.00 | 28.00 | 1.00 | 45.00 | 15.00 |
| A238 | 0.00 | 1.00 | 13.00 | 18.00 | 29.00 | 13.00 | 18.00 | 18.00 | 22.00 | 2.00 | 59.00 | 45.00 |
| A239 | 0.00 | 4.00 | 10.00 | 16.00 | 25.00 | 14.00 | 16.00 | 21.00 | 17.00 | 2.00 | 40.00 | 29.00 |
| A240 | 0.00 | 3.00 | 12.00 | 19.00 | 27.00 | 12.00 | 19.00 | 24.00 | 29.00 | 2.00 | 39.00 | 25.00 |
| A241 | 1.00 | 3.00 | 14.00 | 17.00 | 24.00 | 16.00 | 15.00 | 20.00 | 24.00 | 1.00 | 55.00 | 37.00 |
| A242 | 6.00 | 3.00 | 13.00 | 22.00 | 32.00 | 17.00 | 18.00 | 22.00 | 29.00 | 1.00 | 52.00 | 15.00 |
| A243 | 1.00 | 4.00 | 8.00 | 19.00 | 21.00 | 12.00 | 16.00 | 18.00 | 23.00 | 2.00 | 60.00 | 48.00 |
| A244 | 9.00 | 4.00 | 16.00 | 25.00 | 30.00 | 19.00 | 21.00 | 19.00 | 28.00 | 2.00 | 58.00 | 17.00 |
| A245 | 4.00 | 3.00 | 15.00 | 23.00 | 27.00 | 14.00 | 17.00 | 23.00 | 28.00 | 1.00 | 60.00 | 20.00 |
| A246 | 2.00 | 3.00 | 17.00 | 23.00 | 29.00 | 18.00 | 18.00 | 25.00 | 32.00 | 2.00 | 35.00 | 23.00 |
| A247 | 0.00 | 2.00 | 10.00 | 16.00 | 22.00 | 13.00 | 19.00 | 20.00 | 23.00 | 1.00 | 38.00 | 27.00 |
| A248 | 2.00 | 3.00 | 11.00 | 12.00 | 23.00 | 11.00 | 10.00 | 18.00 | 18.00 | 1.00 | 49.00 | 33.00 |
| A249 | 2.00 | 4.00 | 16.00 | 21.00 | 25.00 | 15.00 | 16.00 | 23.00 | 28.00 | 2.00 | 43.00 | 20.00 |
| A250 | 0.00 | 3.00 | 15.00 | 19.00 | 27.00 | 13.00 | 17.00 | 22.00 | 28.00 | 1.00 | 36.00 | 16.00 |
| A251 | 5.00 | 3.00 | 12.00 | 20.00 | 26.00 | 14.00 | 17.00 | 20.00 | 25.00 | 2.00 | 41.00 | 18.00 |
| A252 | 1.00 | 3.00 | 12.00 | 18.00 | 22.00 | 15.00 | 17.00 | 21.00 | 24.00 | 1.00 | 47.00 | 21.00 |
| A253 | 1.00 | 3.00 | 15.00 | 23.00 | 28.00 | 16.00 | 20.00 | 22.00 | 29.00 | 1.00 | 47.00 | 40.00 |
| A254 | 1.00 | 3.00 | 13.00 | 21.00 | 28.00 | 16.00 | 18.00 | 21.00 | 26.00 | 2.00 | 37.00 | 23.00 |
| A255 | 4.00 | 3.00 | 13.00 | 24.00 | 32.00 | 18.00 | 20.00 | 21.00 | 28.00 | 1.00 | 39.00 | 21.00 |
| A256 | 1.00 | 2.00 | 16.00 | 22.00 | 30.00 | 16.00 | 19.00 | 21.00 | 36.00 | 2.00 | 42.00 | 29.00 |
| A257 | 3.00 | 3.00 | 11.00 | 17.00 | 22.00 | 11.00 | 14.00 | 16.00 | 26.00 | 2.00 | 45.00 | 26.00 |
| A258 | 3.00 | 3.00 | 11.00 | 19.00 | 29.00 | 17.00 | 20.00 | 23.00 | 31.00 | 2.00 | 38.00 | 30.00 |
| A259 | 0.00 | 2.00 | 14.00 | 24.00 | 28.00 | 17.00 | 19.00 | 21.00 | 26.00 | 2.00 | 60.00 | 40.00 |
| A260 | 0.00 | 4.00 | 6.00 | 14.00 | 16.00 | 9.00 | 8.00 | 19.00 | 12.00 | 2.00 | 34.00 | 18.00 |

| | | | | | | | | | | | | |
|---|---|---|---|---|---|---|---|---|---|---|---|---|
| A261 | 6.00 | 3.00 | 19.00 | 30.00 | 27.00 | 22.00 | 10.00 | 23.00 | 27.00 | 2.00 | 38.00 | 20.00 |
| A262 | 3.00 | 3.00 | 15.00 | 23.00 | 23.00 | 14.00 | 17.00 | 20.00 | 31.00 | 1.00 | 33.00 | 15.00 |
| A263 | 5.00 | 2.00 | 18.00 | 26.00 | 33.00 | 19.00 | 20.00 | 25.00 | 36.00 | 1.00 | 44.00 | 15.00 |
| A264 | 2.00 | 2.00 | 15.00 | 27.00 | 31.00 | 18.00 | 19.00 | 24.00 | 33.00 | 1.00 | 52.00 | 27.00 |
| A265 | 1.00 | 2.00 | 11.00 | 18.00 | 24.00 | 10.00 | 13.00 | 12.00 | 18.00 | 1.00 | 35.00 | 20.00 |
| A266 | 1.00 | 3.00 | 12.00 | 18.00 | 22.00 | 15.00 | 17.00 | 21.00 | 24.00 | 1.00 | 45.00 | 35.00 |
| A267 | 2.00 | 2.00 | 12.00 | 17.00 | 23.00 | 12.00 | 15.00 | 18.00 | 24.00 | 2.00 | 35.00 | 16.00 |
| A268 | 2.00 | 3.00 | 17.00 | 18.00 | 24.00 | 14.00 | 10.00 | 15.00 | 22.00 | 2.00 | 43.00 | 16.00 |
| A269 | 1.00 | 3.00 | 11.00 | 18.00 | 24.00 | 10.00 | 13.00 | 12.00 | 18.00 | 1.00 | 31.00 | 23.00 |
| A270 | 2.00 | 3.00 | 12.00 | 18.00 | 24.00 | 13.00 | 17.00 | 19.00 | 24.00 | 1.00 | 53.00 | 20.00 |
| A271 | 1.00 | 3.00 | 12.00 | 17.00 | 26.00 | 13.00 | 19.00 | 20.00 | 25.00 | 1.00 | 40.00 | 34.00 |
| A272 | 4.00 | 1.00 | 12.00 | 26.00 | 28.00 | 17.00 | 20.00 | 16.00 | 28.00 | 2.00 | 37.00 | 22.00 |
| A273 | 1.00 | 2.00 | 12.00 | 18.00 | 22.00 | 12.00 | 15.00 | 18.00 | 24.00 | 2.00 | 47.00 | 20.00 |
| A274 | 1.00 | 2.00 | 15.00 | 25.00 | 25.00 | 16.00 | 17.00 | 21.00 | 27.00 | 2.00 | 40.00 | 23.00 |
| A275 | 1.00 | 3.00 | 7.00 | 11.00 | 16.00 | 6.00 | 17.00 | 14.00 | 15.00 | 1.00 | 41.00 | 28.00 |
| A276 | 3.00 | 4.00 | 12.00 | 21.00 | 25.00 | 17.00 | 18.00 | 17.00 | 23.00 | 1.00 | 50.00 | 23.00 |
| A277 | 1.00 | 3.00 | 11.00 | 19.00 | 25.00 | 12.00 | 18.00 | 19.00 | 26.00 | 1.00 | 58.00 | 36.00 |
| A278 | 5.00 | 2.00 | 10.00 | 17.00 | 20.00 | 13.00 | 19.00 | 21.00 | 23.00 | 2.00 | 31.00 | 21.00 |
| A279 | 3.00 | 3.00 | 15.00 | 19.00 | 27.00 | 13.00 | 17.00 | 20.00 | 27.00 | 1.00 | 40.00 | 22.00 |
| A280 | 4.00 | 1.00 | 16.00 | 24.00 | 24.00 | 24.00 | 23.00 | 20.00 | 35.00 | 1.00 | 56.00 | 32.00 |
| A281 | 2.00 | 2.00 | 19.00 | 24.00 | 31.00 | 17.00 | 22.00 | 23.00 | 34.00 | 1.00 | 49.00 | 12.00 |
